# Disparities of obesity and non-communicable disease burden between the Tibetan Plateau and developed megacities in China

**DOI:** 10.3389/fpubh.2022.1070918

**Published:** 2023-01-10

**Authors:** Wen Peng, Wenxiu Jian, Tiemei Li, Maureen Malowany, Xiao Tang, Mingyu Huang, Youfa Wang, Yanming Ren

**Affiliations:** ^1^Nutrition and Health Promotion Center, Department of Public Health, Medical College, Qinghai University, Xining, China; ^2^Qinghai Provincial Key Laboratory of Traditional Chinese Medicine Research for Glucolipid Metabolic Diseases, Medical College, Qinghai University, Xining, Qinghai, China; ^3^Faculty of Medicine, Braun School of Public Health and Community Medicine, Hebrew University of Jerusalem—Hadassah Medical Organization, Jerusalem, Israel; ^4^Medical College, Qinghai University, Xining, China; ^5^Global Health Institute, School of Public Health, Xi'an Jiaotong University, Xi'an, China

**Keywords:** obesity, non-communicable diseases, disparities, socioeconomic status, dietary intakes

## Abstract

**Background:**

Non-communicable diseases (NCDs) including risk factors, e.g., obesity, are the major causes of preventable deaths in China, yet NCD disparities in China remain under-studied.

**Objective:**

This study aimed to compare the determinants and burden of NCDs within four selected provinces in mainland China: the least developed Qinghai-Tibet Plateau group (PG, Tibetan Autonomous Region [TAR] and Qinghai Province) and most developed megacity group (MCG, Shanghai, and Beijing).

**Methods:**

Studies, reports, and other official sources with comparable data for NCD burden and related determinants for the four provinces were searched. Geographic, demographic, socioeconomic, and dietary characteristics and selected health indicators (e.g., life expectancy) were extracted from the China Statistical Yearbook and China Health Statistics Yearbook. Data on NCD burdens were extracted from the National Chronic Disease and Risk Factor Surveillance Study and other nationally representative studies.

**Results:**

The overall NCD mortality rates and prevalence of metabolic risk factors including obesity, hypertension, and diabetes in mainland China have increased in the past 20 years, and this trend is expected to continue. The PG had the highest level of standardized mortality rates (SMRs) on NCDs (711.6–896.1/100,000, 6th/6-level); the MCG had the lowest (290.6–389.6/100,000, 1st/6-level) in mainland China. The gaps in SMRs were particularly high with regard to chronic respiratory diseases (PG 6th^/^6-level, MCG 1st/6-level) and cardiovascular diseases (6th/6 and 4th/6 in TAR and Qinghai; 1st/6-level and 2nd/6-level in Shanghai and Beijing). In contrast, the prevalence rates of obesity, hypertension, and diabetes were generally higher or comparable in MCG compared to PG. Diabetes prevalence was particularly high in MCG (5th/5-level, 13.36–14.35%) and low in PG (1st/5-level, 6.20–10.39%). However, awareness, treatment, and control of hypertension were poor in PG. Additionally, PG had much lower and severely inadequate intakes of vegetables, fruits, and dairy products, with additional indicators of lower socioeconomic status (education, income, etc.,) compared with MCG.

**Conclusion:**

Evidence showed large disparities in NCD burden in China's provinces. Socioeconomic disparity and dietary determinants are probably the reasons. Integrated policies and actions are needed.

## 1. Introduction

Reducing health disparities is a priority set by the Sustainable Development Goals of the United Nations (1) and by the governments of many countries including China, reinforced with the adoption of the Healthy China Initiative 2030 (2). Health disparities are the result of many complex factors and their interactions at all population levels from the individual, community, and provincial to national (3). Health disparities globally and in China are evidenced across areas and populations at various socioeconomic development levels and, in addition, have a direct relationship to the ultimate health outcome of life expectancy (LE). Globally, the range of this gap in LE in 2020 was as high as 30 years (Japan 84.6 years and Central African Republic 53.7 years) (4). Within China, the gap between two selected provinces, the most and least developed, respectively, is significant (Shanghai 84.1 vs. Tibetan Autonomous Region (TAR) 71.1 years) (5–9). Among the many factors affecting LE, non-communicable diseases (NCDs) are considered among the most worrying, and playing increasing important roles.

Non-communicable diseases present major disease burdens worldwide contributing to 71% of global mortality (10). In China, deaths due to NCDs increased from 72% in 1990 to almost 90% in 2019 (11). Globally, almost three-quarters of all NCD deaths and 82% of the 16 million people who died prematurely (before reaching 70 years of age) occurred in low- and middle-income countries (LMICs) (12). Previous studies showed low socioeconomic status (SES) and/or living in LMICs increased the risk of developing particular NCDs, e.g., cardiovascular diseases, lung and gastric cancer, Type 2 diabetes, and chronic obstructive pulmonary disease (COPD). Moreover, low SES increased the risk of mortality from some NCDs (13). Given the high NCD burden in China and the evidence of prevailing NCD disparities globally, it is necessary to examine with attention to local conditions or risk factors or health determinants carefully. An assessment of disparities at the provincial level will facilitate meeting the targets of both the Healthy China Initiative and Sustainable Development Goals.

Studies on NCD disparities in China are limited in number and scope. Studies focused on the health service utilization of NCDs revealed increased access to NCD care for the wealthier population (14–16). A recent study compared national urban/rural health expenditure on NCDs (17). However, we did not find any study that comprehensively investigated the disparities in the prevalence, treatment, and related risk factors of common NCDs in China's provinces.

As an entry point to study this broad topic, we selected four provincial-level administrative units (we will use the term province consistently in this manuscript) primarily based on their socioeconomic development. Other factors such as geographic features, literacy level, urbanization rates, and the proportion of minority populations were also taken into consideration. Based on the above selection principles and relevant to our team's interest in the Qinghai-Tibet Plateau, we selected four provinces, i.e., Qinghai, TAR, Beijing, and Shanghai, which were further divided into two groups. Qinghai and the TAR located on the Qinghai-Tibet Plateau formed the Plateau group (PG), while Beijing and Shanghai formed the megacity group (MCG).

The Qinghai-Tibet Plateau has an average altitude of more than 4,500 m above sea level with a sizeable population of minority groups such as Tibetans, Muslims, and others. The unique hypobaric hypoxia environment and other related factors such as suboptimal socioeconomic development may have caused the lowest level of LE in China, compared with the highest level of LE in developed megacities. In 2020, the LE for the TAR, Qinghai, Shanghai, and Beijing was 71.1, 73.7, 84.1, and 82.4 years, respectively (6–9).

NCD burdens probably have contributed largely to the health disparity between the Plateau and developed areas. Our recent field study and meta-analysis showed a high prevalence of obesity, hypertension, and dyslipidemia, the metabolic risk factors of NCDs, among Tibetan adults on the Qinghai-Tibet Plateau (18–20). However, we did not find any study that investigated the NCD disparities between residents of the Qinghai-Tibet Plateau and those in developed areas.

To rectify this evidence gap, the present study will compare the NCD burdens and their potential determinants between the Qinghai-Tibet Plateau and developed megacities in China. This research will provide significant insights to address NCD and health disparities in China.

## 2. Methods

This study followed the World Health Organization (WHO) NCD 5^*^5 framework and investigated the major NCD burdens in mainland China and, based on data availability, their potential lifestyle and metabolic risk factors.

### 2.1. Case selection

We selected four provincial-level administrative areas in mainland China to form two comparative groups. The PG consists of the TAR and Qinghai Province, which has the lowest LE in mainland China. The developed MCG consists of Beijing and Shanghai, which has the highest LE in mainland China. We also investigated the trends of the overall NCD burden in mainland China to have a broader contextual understanding at the national level.

**Figure 1:**
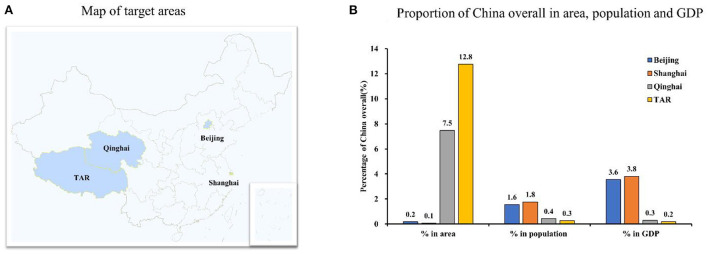
Comparison of Beijing, Shanghai, Qinghai and Tibetan Autonomous Region in geographic location, area, population and GDP in 2021. TAR, Tibet Autonomous Region; GDP, Gross Domestic Products. **(A)** Map of target areas. **(B)** Proportion of China overall in area, population and GDP. Data were from the Statistical Yearbook of China (2021).

The four selected provinces have distinct features in terms of geographic, demographic, and socioeconomic characteristics. Figure 1 shows that the PG (the TAR and Qinghai) are extremely large in area, very small in population, and low in GDP. The TAR accounted for 12.8% of China's total area but only 0.3% of the population and 0.2% of GDP. The proportions for Qinghai were 7.5, 0.4, and 0.3%, respectively. For the MCG, Beijing, and Shanghai, the areas are relatively small while populations and GDPs relatively large.

The geographic, demographic, and socioeconomic characteristics of the four provinces in 2021 are further compared in Table 1. The PG features by being high in elevation above the sea level (2,262 and 3,650 m in elevation for the capital of Qinghai and TAR, respectively) and a significant percentage of minority populations (49.5% for Qinghai and 87.9% for TAR). In Qinghai, Tibetan and Muslims are the major minority populations, while the Tibetan population alone accounts for the majority in the TAR. The PG had below the national average and much lower levels in all the socioeconomic indicators in Table 1 than the MCG. In particular, the illiteracy rate in the TAR was almost 20 times higher than that in Beijing.

**Table 1:** Comparison of geographic, demographic, and socioeconomic characteristics of Beijing, Shanghai, Qinghai, TAR, and mainland China overall.

	**Geographic**			**Demographic** ^**3**^		**Socioeconomic** ^**3**^				
	**Region**	**Altitude (m)** ^1^	**Area (1,000 km** ^2^ **)**	**Population (million)**	**% Of minority population**	**GDP (billion Yuan)** ^2^	**GDP per capita (1,000 Yuan)**	**Urbanization rate (%)**	**Illiteracy rate (%)**	**Disposabal income per capita (1,000 Yuan)**
China Mainland		1,840	9,638	1,409.8	8.9	101,598.6	72.0	63.9	4.6	
Beijing	North	43.5	17	21.9	4.8	3,610.3	164.9	87.6	1.7	69.4
Shanghai	East	2.19	6	24.9	1.6	3,870.1	155.8	89.3	2.5	72.2
Qinghai	Northwest	2,262 (Xining)	720	5.9	49.5	300.6	50.8	60.1	10.6	24
TAR	Southwest	3,650 (Lhasa)	1230	3.6	87.9	190.3	52.3	35.7	33.1	21.7

1 The altitude of Qinghai: 1,650–6,860 m; the altitude of TAR: 2,500–8,000 m.

2 Notably, 100 Chinese Yuan is equal to 15.13 USD.

3 Demographic and socioeconomic data came from the China Statistical Yearbook (2021).

TAR, Tibet Autonomous Region.

### 2.2. Literature search strategies

We searched and reviewed all published national survey reports and original studies in academic journals conducted by the national Chinese Diseases Control and Prevention Center (CDC) and provincial CDCs of the four selected provinces for data related to the burden of NCDs in China and in the four provinces, respectively, over the past 15 years. Other representative national surveys, published between January 2008 and April 2022, which included the four target provinces were also searched and reviewed. Keywords used in our search on PubMed, CNKI (China National Knowledge Infrastructure), and Wanfang databases included “China,” “Beijing,” “Shanghai,” “Qinghai,” “Tibetan,” “NCDs,” “non-communicable diseases,” “hypertension,” “diabetes,” “mortality,” “standardized mortality,” “SMR,” “prevalence,” “management,” “awareness,” “treatment,” and “control.” The references of significant publications were also cross-checked for potential data sources. Statistics Yearbooks and official governmental webpages were searched for related information for China overall and the four selected provinces individually and collectively.

### 2.3. Inclusion criteria

The inclusion criteria were as follows: (1) data collection conducted by the Chinese CDC system (including the provincial CDCs) using nationally or provincially representative samples; (2) published reports or studies from other nationally or provincially representative surveys; (3) studies providing data on NCD burdens in China overall and the four selected provinces; and (4) surveys in which the participants were aged ≥18 years. Finally, from the literature searched, considering data comparability, we included data from seven national survey reports and the latest four province official websites published data. Seven national survey reports are China Statistical Yearbook (2021) (5), China Health Statistics Yearbook (2021) (21), Report on Chronic Disease Risk Factor Surveillance in China (CCDRFS) (2002, 2013, 2018) (22–24), and Report on Chinese Residents' Chronic Diseases and Nutrition (2015, 2020) (11, 25).

### 2.4. Key variables and data source

#### 2.4.1. General characteristics and selected health indicators

The data on general characteristics including the area, population, and Gross Domestic Products (GDP) of the four selected provinces were obtained from the China Statistical Yearbook (2021) (5).

Selected health indicators of the four provinces were extracted from the China Health Statistics Yearbook (2021) (21) (LE, health technicians per 1,000 people and beds in healthcare facilities per 1,000 people) and provincial government official websites (health literacy rates) (6–9, 26).

#### 2.4.2. NCD burden

The standardized mortality rates (SMRs) due to major NCDs in the four provinces were derived from the Report on Nutrition and Chronic Disease Status Survey of Chinese Residents (2020). The SMRs were calculated using the standard population constituent ratio and actual age-specific mortality rate. The world population structure in 2011 was established as the standard population (11). Data on the prevalence of obesity, central obesity, and hypertension and control of hypertension were from the CCDRFS studies (2013 survey)(27). We did not find published comparable data on the control of diabetes for the four provinces. Due to the unavailability of recent data in CCDRFS studies, diabetes prevalence was derived from another nationally representative survey conducted in 2015–2017 (28).

Published data on NCD burdens, including the mortality rates, prevalence, and control rates, by provinces, were expressed with ordinal categorical variables with a reference range for each category. Thus, the NCD burdens were shown as ordinal but not continuous variables in this study.

#### 2.4.3. Potential determinants of NCD burden

Demographic, socioeconomic, and dietary characteristics of the four provinces were extracted from the China Statistical Yearbook (2021) (5).

Demographic indicators included the total population and proportion of ethnic minorities. Socio-economic indicators included GDP, GDP per capita, illiteracy rate, urbanization rate, and disposable income per capita. Food consumption data included the following six food categories: (1) cereals; (2) meat, poultry, aquatic products, and eggs; (3) milk and dairy products; (4) fruits; (5) edible oil; and (6) vegetables and edible mushrooms. The proportional structure of food types in the second category was further analyzed.

### 2.5. Statistical analysis

**Figure 2:**
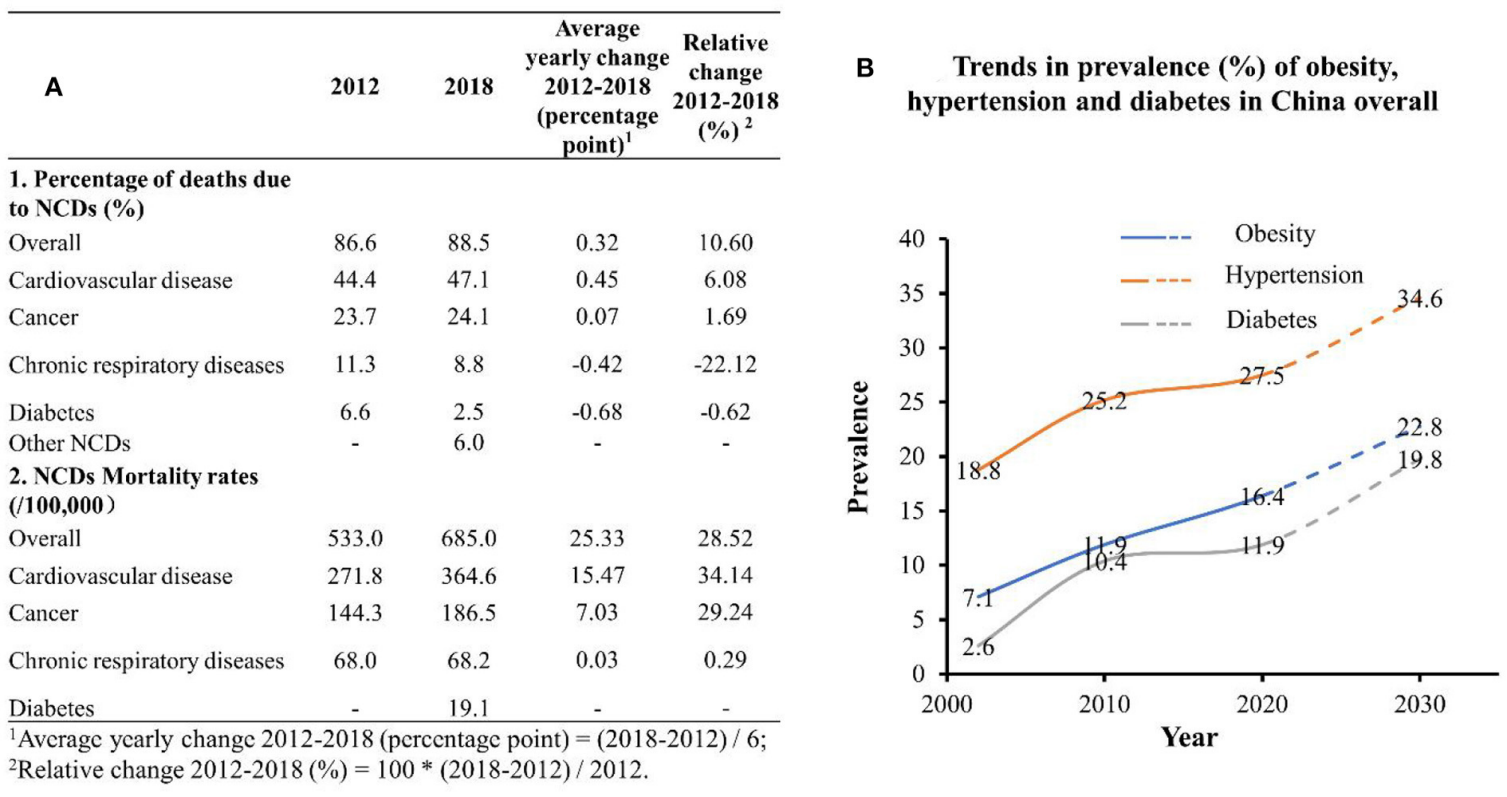
Trends in major NCD mortality and prevalence in China overall, 2002–2030. **(A)** Data were from the Report on Nutrition and Chronic Disease Status of Chinese Residents (2015) and the Report on Nutrition and Chronic Disease Status of Chinese Residents (2020); **(B)** data were from the China Chronic Disease and Risk Factor Surveillance Report (2013), Report on Nutrition and Chronic Disease Status of Chinese Residents (2015), and the Report on Nutrition and Chronic Disease Status of Chinese Residents (2020). NCD, non-communicable disease.

To describe NCD mortality trends in China overall, we calculated the average yearly change and relative change from 2012 to 2018 in attributed proportional deaths and mortality rates due to NCDs. The calculating method is described in detail in Figure 2, footnote.

Trends in the prevalence of obesity, hypertension, and diabetes in China overall from 2002 to 2018 were described. Linear regression models were used to predict the three prevalence rates in 2030. The goodness-of-fit (R^∧^2) was used to evaluate how well the regression line fit observed values.

To compare the NCD burden in selected provinces in China, this review study extracted the related comparable indicators from available public sources, e.g., peer-reviewed journals, official reports, and other official data sources. We compared the indicators directly to highlight NCD disparities. In addition, we have made extensive efforts to look for the indicators across different years, in order to compare the indicators among selected provinces. However, no NCD burden indicators were available for the PG (Qinghai and the TAR). To address this gap, we compared the extracted indicators of NCD burden directly. From a public health perspective, the disparity in data availability between groups highlighted the multifaceted efforts needed to examine and decrease health disparities in different areas in China.

## 3. Results

### 3.1. Overall NCD burden in China

To have a broad understanding of NCD burdens in China for a more exhaustive comparison, we investigated the trends of overall NCD burdens at the national level. The burden of NCD in China was relatively high and is expected to continue to increase. As shown in Figure 2, the proportion of NCD deaths as a percentage of national deaths and NCD mortality rates (/100,000) increased continuously from 2012 to 2018. The prevalence rates of obesity, hypertension, and diabetes in China overall also increased from 2002 to 2018, and reached 16.4, 27.5, and 11.9% in 2018, respectively. The rates were projected to reach 22.8, 34.6, and 19.8%, respectively, in 2030.

### 3.2. Trends of selected health indicators

**Figure 3:**
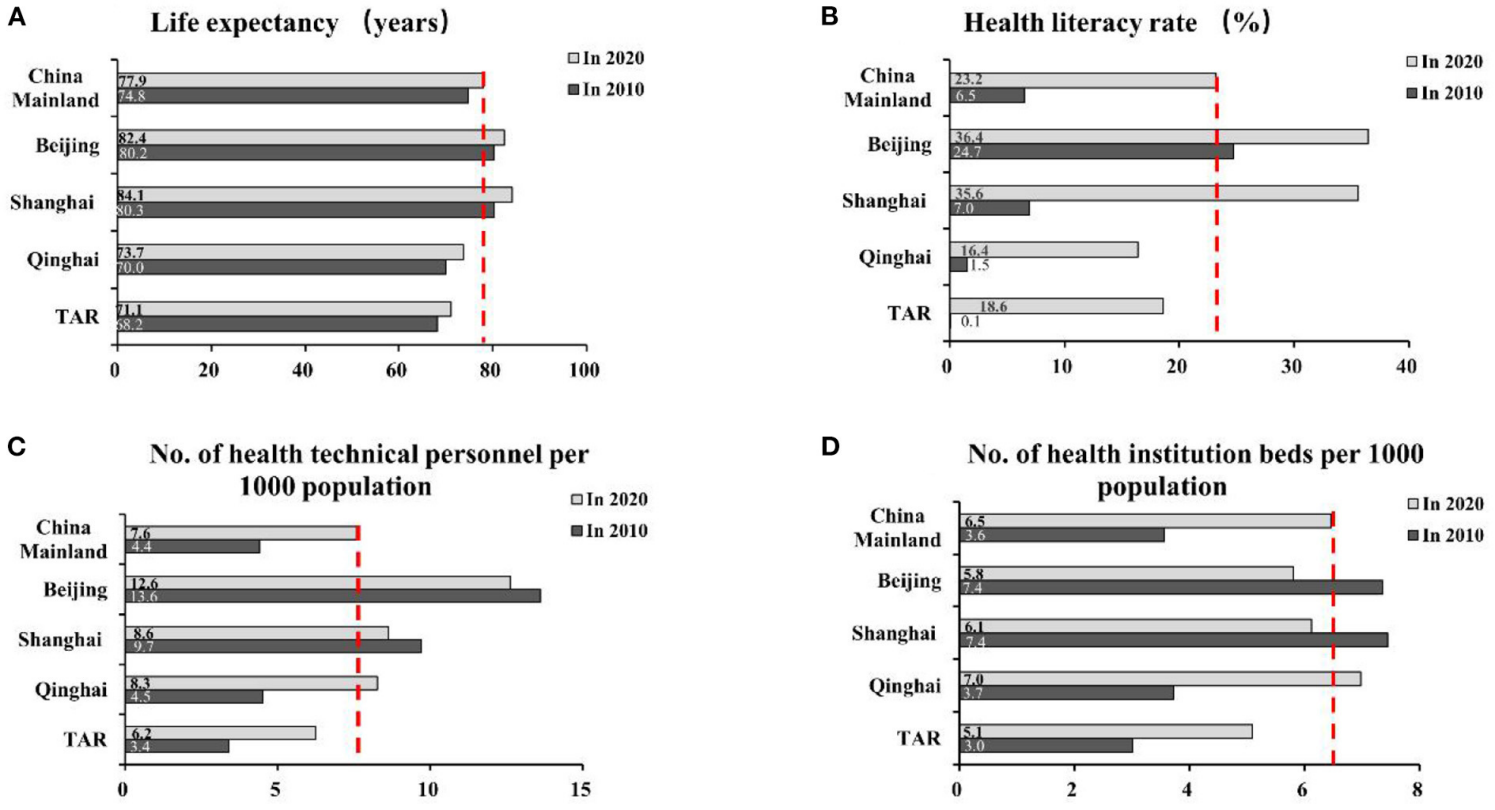
Selected health indicators in Beijing, Shanghai, Qinghai, and TAR in comparison with mainland China overall, 2010 and 2020. TAR, Tibet Autonomous Region; dotted line indicates National average; **(A, C, D)** Data were from China Health Statistics Yearbook (2021); **(B)** data were from the official webpages of the four provincial and municipal governments (2021).

Some selected health indicators and their trends over the past decade are compared in Figure 3. Data show the LE and health literacy rate in all four provinces and in China overall increased during this period, while the gaps between groups remained in 2020. In 2020, the LE in Shanghai was 13 years higher than that in the TAR (84.1 vs. 71.1 years), and the health literacy rates in the PG were still approximately half of those in the MCG. In contrast, the PG's sharp increase in the number of health technical personnel and number of health institution beds per 1,000 populations and the narrowing intergroup difference showed strides in health infrastructure in the past decade.

### 3.3. SMRs due to NCDs

**Figure 4:**
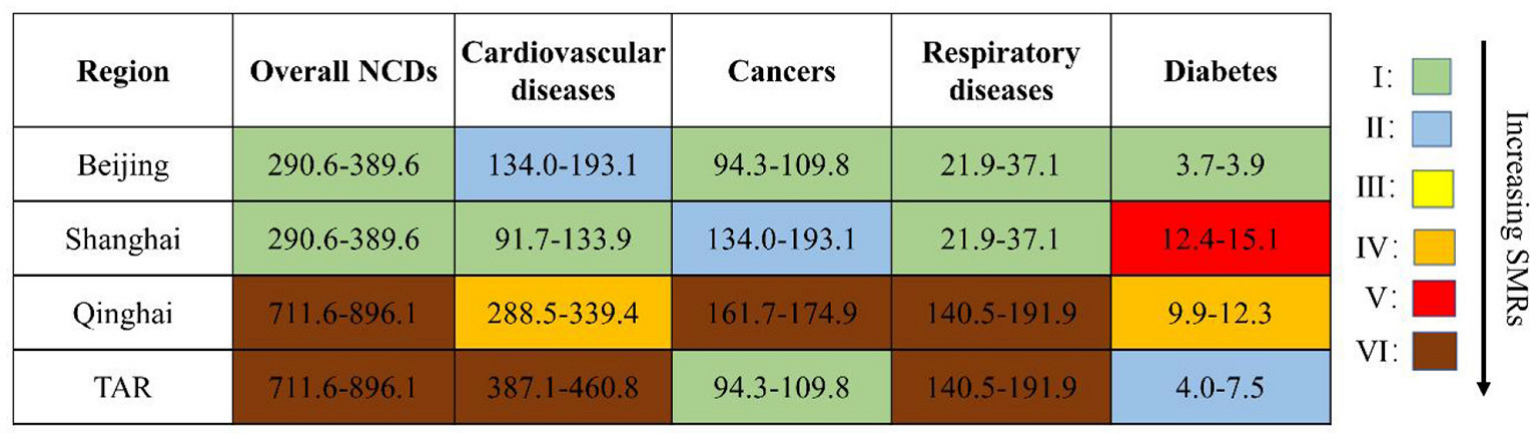
Comparison of SMRs due to four major NCDs in Beijing, Shanghai, Qinghai, and TAR in 2019 (deaths per 100,000 population). Overall NCDs: I: 290.6–389.6; II: 389.7–494.0; III: 494.1–595.1; IV: 595.2–651.7; V: 651.8–711.5; VI: 711.6–896.1. Cardiovascular diseases: I: 91.7–133.9; II: 134.0–193.1; III: 193.2–288.4; IV: 288.5–339.4; V: 339.5–386.9; VI: 387.1–460.8. Cancers: I: 94.3–109.8; II: 109.9–124.5; III: 124.6–134.9; IV: 135.0–146.6; V: 146.7–161.6; VI: 161.7–174.9. Respiratory diseases: I: 21.9–37.1; II: 37.2–49.9; III: 50.0–58.8; IV: 58.9–88.6; V: 88.7–140.4; VI: 140.5–191.9. Diabetes: I: 3.7–3.9; II: 4.0–7.5; III: 7.6–9.8; IV: 9.9–12.3; V: 12.4–15.1; VI: 15.2–19.7. SMR, standardized mortality rate; TAR, Tibet Autonomous Region; NCD, non-communicable disease. Data were from the Report on Chronic Disease Risk Factor Surveillance in China (2020).

The SMRs due to NCDs overall and four major NCDs in the four target provinces in 2019 were compared by ordinal categories in Figure 4. The PG shows the highest SMRs on NCDs overall (711.6–896.1/100,000, 6^*th*^/6-level), while the MCG had the lowest (290.6–389.6/100,000, 1^*st*^/6-level) in mainland China. The gaps in SMRs were particularly high for chronic respiratory diseases (6^*th*^/6-level in TAR and Qinghai, 1^*st*^/6-level in Shanghai and Beijing) and cardiovascular diseases (6^*th*^ and 4^*th*^/6-level in TAR and Qinghai, respectively; 1st and 2^*nd*^/6-level in Shanghai and Beijing, respectively). Of note, the SMR due to cancers in Qinghai was in the highest category (6^*th*^/6-level), and the SMR due to diabetes in Shanghai was also unexpectedly high (4^*th*^/6-level).

### 3.4. Prevalence and control of metabolic risk factors of NCDs

**Figure 5:**
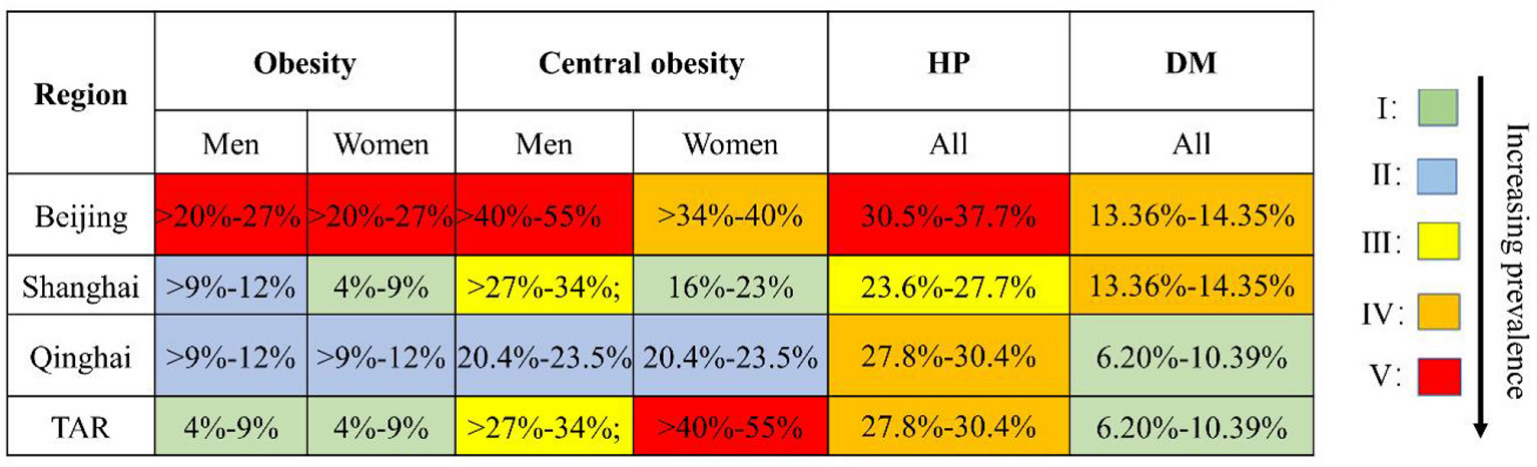
Comparison of prevalence of obesity, central obesity, hypertension, and diabetes in Beijing, Shanghai, Qinghai, and TAR, 2013–2017. Obesity: I: 4–9%; II: >9–12%; III: >12–14%; IV: >14–20%; V: >20–27%. Central obesity: I: 16–23%; II:>23–27%; III: >27–34%; IV: >34–40%; V: >40–55%. Hypertension: I: 18.0–20.3%; II: 20.4–23.5%; III: 23.6–27.7%; IV: 27.8–30.4%; V: 30.5–37.7%. Diabetes: I: 6.20–10.39%; II: 10.64–11.93%; III: 11.99–12.90%; IV: 13.36–14.35%; V: 14.38–19.85%. TAR, Tibet Autonomous Region; HP, hypertension; DM, diabetes. The prevalence of obesity, central obesity, and hypertension was from China Chronic Disease and Risk Factor Surveillance Report (2013). The prevalence of diabetes was from a nationally representative study conducted in 2015–2017.

The prevalence of metabolic risk factors of NCDs in Figure 5 in Beijing was the highest among the four provinces. Central obesity among women was an exception. The PG recorded high hypertension (27.8–30.4%, 4th/5-level) and low diabetes (6.20–10.39%, 1st/5-level) prevalence. The high prevalence of central obesity among TAR women (40.1–55.0%, 5th/5-level) was an outlier (Figure 5).

**Figure 6:**
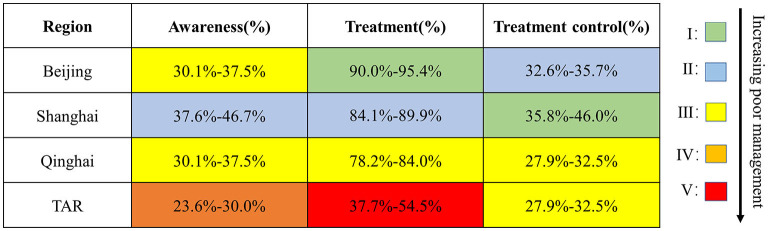
Awareness: V: 18.0%–23.5%; IV: 23.6%–30.0%; III: 30.1%–37.5%; II: 37.6%–46.7%; I: 46.8%–56.1%. Treatment: V: 37.7%–54.5%; IV: 54.6%–78.1%; III: 78.2%–84.0%; II: 84.1%–89.9%; I: 90.0%-95.4%. Treatment control: V: 15.1%-19.6%; IV: 19.7%-27.8%; III: 27.9%-32.5%; II: 32.6%–35.7%; I: 35.8%–46.0%. TAR, Tibet Autonomous Region. Data were from the Report on Chronic Disease Risk Factor Surveillance in China (2013).

Among the three indicators of hypertension management in Figure 6, data for the PG demonstrated overall poor performance. The TAR had comprehensively the lowest percentage and Shanghai had the highest of the four provinces. To the best of our knowledge, representative data on diabetes management for the four provinces were not publicly available.

### 3.5. Food consumption characteristics

**Figure 7:**
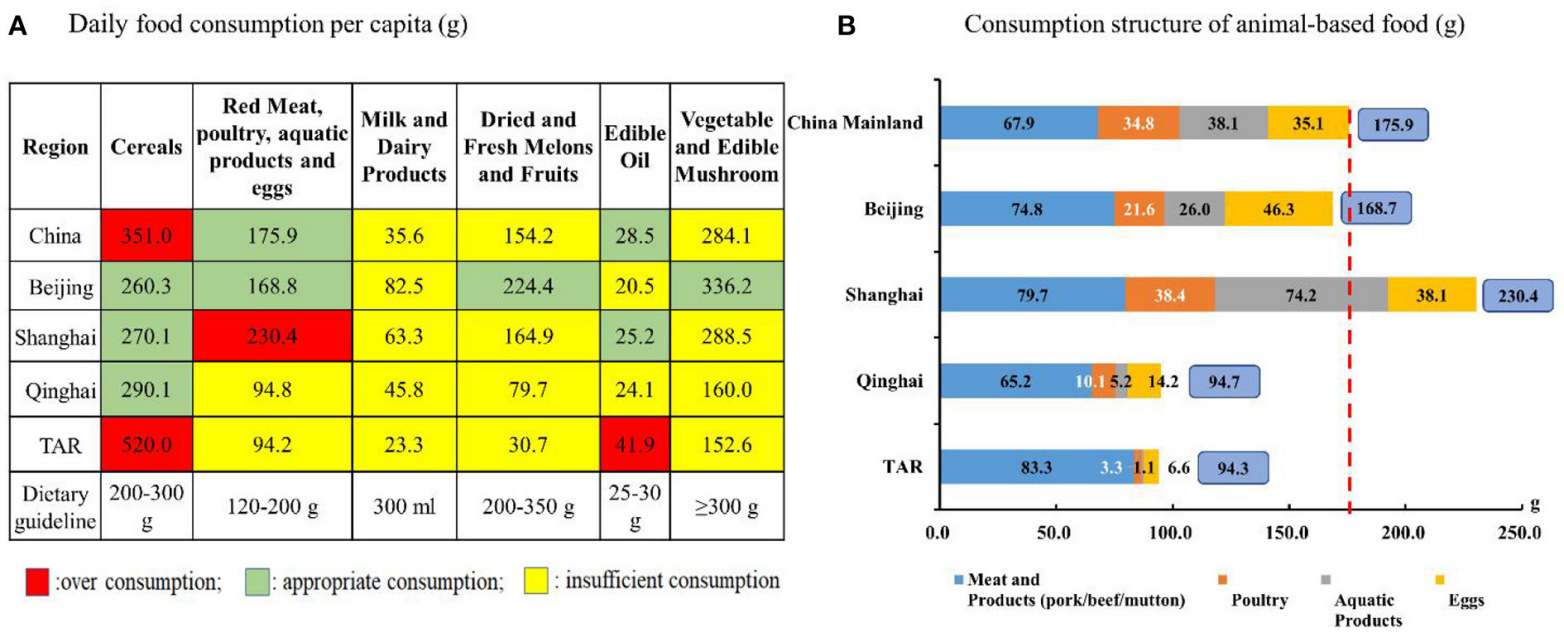
Daily food consumption per capita in Beijing, Shanghai, Qinghai, and TAR in comparison with China overall in 2021. The dotted line indicates the National average. TAR, Tibet Autonomous Region. **(A, B)** Data were from the Statistical Yearbook of China (2021).

The intergroup differences in daily food consumption per capita were tremendous (Figure 7). The PG had severely inadequate and much lower intakes of vegetables, fruits, and dairy products and an inadequate total of animal-based food (dairy excluded). In the proportional structure of animal-based food intakes, in comparison with Beijing and Shanghai with reported < 45% red meat consumption among animal-based food, Qinghai and TAR reported relatively large percentages of red meat consumption (68.8 and 87.9%, respectively).

## 4. Discussion

This study demonstrated that the Qinghai-Tibet Plateau, when compared with developed megacities in mainland China, had much higher SMRs due to NCDs, higher hypertension prevalence, and poorer awareness, treatment, and control of hypertension. These data support the suggested high burden of NCDs on the Qinghai-Tibet Plateau. With reference to the comparative description of socioeconomic status and lifestyle factors, we postulated that the comprehensive health outcome of the higher/highest SMRs due to NCDs for the PG resulted from the complex interaction of many factors, such as poor NCD management, low socioeconomic status (education, income, etc.), and prevalent behavioral risk factors (e.g., unhealthy diets). In addition, the intragroup differences in the pattern of NCD burdens were also interesting.

This study initially showed the comprehensive NCD disparities between Plateau residents and those living in developed megacities. The identified potential determinants should be the targets to prevent premature deaths due to NCDs and to reduce further NCDs and health disparities.

### 4.1. Intergroup comparison

The much higher NCD burdens in the PG suggest an association with low socioeconomic status, the high-altitude hypobaric environment, and the prevalent and increased behavioral risk factors.

Evidence in high-income and transitional countries has shown socioeconomically disadvantaged populations that have been disproportionately affected by premature death due to NCDs (29). Even worse, NCDs may exacerbate the economic status among the poor by increasing healthcare-related costs and decreasing the labor capability of patients with NCDs (30). A nationwide survey of 29,712 rural poor households in China showed 51.63% of households attributed their poverty to illness mostly NCDs (31). Reciprocally, NCDs were also important reasons for recurring poverty (30). The unbalanced distribution of high-quality healthcare resources in China was another key contributor to the NCD disparity (17, 32, 33). Accessibility to high-quality healthcare in the very remote pastoral areas on the Plateau was also challenging (34).

Findings from the intergroup comparison in this study suggested potential ethnic disparity in NCD burden, which was consistent with other studies (28, 35–39). Data for populations in the United States have shown that Non-Hispanic black and Hispanic adults had a higher prevalence of obesity and diabetes than those recorded for non-Hispanic white adults (35, 36). In China, several nationally representative surveys have shown consistent gaps in the prevalence and management rates of common NCDs, such as hypertension and diabetes among ethnic groups (28, 37–39). In this study, the high proportion of minority populations in Qinghai (almost 50%, mainly Tibetan and Muslim) and TAR (almost 90%, mainly Tibetan) and the corresponding high NCD burdens were consistent with previous findings. The underlying reasons and the coping strategies for the ethnic disparity in NCDs need to be explored.

The particular high SMRs due to cardiovascular and chronic respiratory diseases in the PG may be explained by the extra stress placed on the cardiovascular and respiratory systems from the hypobaric hypoxia environment. Our recently published study showed higher hypertension prevalence among Tibetans, who usually reside on the Plateau, as compared with China's national average (18, 19, 40). Another earlier study also showed a positive association between altitude and hypertension. A 2% increase in hypertension prevalence with every 100 m increase in altitude was revealed (41). Further evidence supported these associations as reported in a systematic review and meta-analysis of 40,854 Tibetans living on the plateau reported an increase in systolic blood pressure of 17 mmHg and diastolic blood pressures of 9.5 mmHg, with an elevation of every 1,000 m (42). In addition, chronic mountain diseases, which affect 5–10% of permanent residents in the highlands, and which can occur in both native and migrated populations on the plateau, may exacerbate related burdens (43–45). The related physiological process includes the progressive loss of ventilatory function, resulting in excessive hypoxemia and erythrocytosis. This syndrome is often associated with pulmonary hypertension and can progress to cardiopulmonary failure (46–48). The excess burden on the cardiopulmonary system by the hypobaric hypoxia environment on the plateau can partially explain the high SMRs.

The unbalanced diets among Plateau residents featured high salt, high edible oil, and low vegetables/fruit intakes, as revealed in this and a previous study (49), were considered a modifiable risk factor for NCDs. This was probably due to the huge area, sparsely populated residents, large health service radius, and poor utilization of health resources on the remote Plateau, despite that Qinghai was catching up with Shanghai in the number of health personnel and health institutional beds in the last decade (50, 51). The very low frequency of whole grain intakes and the preference for red meat (e.g., beef and mutton) among residents on the Plateau, as shown in this and our previous studies (19), were suggested to be associated with an increased risk of incident cardiovascular diseases and all-cause mortality (52, 53). We suggest that these dietary characteristics among the PG have contributed to the NCD disparities as compared with the MCG. Promoting healthy diets was proved to result in the largest economic return based on the WHO estimates (54). Culturally adapted and population-tailored intervention studies need to be developed.

It should be noted that the gap in LE between the lowest in TAR and the highest in Shanghai has enlarged from 2010 to 2020. Urgent actions are needed to prevent increasing regional health disparities.

### 4.2. Intragroup comparison

In the MCG, the much higher prevalence of obesity, hypertension, and diabetes in Beijing in the North compared to Shanghai in the South was consistent with other nationally representative studies (28, 55, 56). More interestingly, the north-south decreasing gradients in average body mass index (BMI) and obesity prevalence were suggested (56). The differences in salt intake and air pollution, the fifth risk factor in the WHO NCD framework, may have also contributed to the NCD differences between Beijing and Shanghai (57, 58). These interesting findings and potential causal associations suggest that regionally tailored intervention strategies are needed.

In the PG, the higher SMR in cardiovascular diseases in TAR compared with Qinghai may be associated with the higher degree of hypobaric hypoxia-induced stress, lower socioeconomic status, and even unhealthier diets (e.g., severe insufficient vegetable and fruit intake), as revealed in this study. The very high SMR in cancers in Qinghai was reported to be associated with esophageal and stomach cancers (59), but underlying factors need to be further explored.

### 4.3. Strengths and limitations

This study has important strengths. The study results provided a comprehensive broad picture of NCD disparities and potential determinants in four selected cases with distinct geographic, demographic, and socioeconomic characteristics. The intergroup and intragroup comparisons provided important insights for the development of tailored NCD interventions with a long-term goal to promote health equity.

This study has also several limitations. First, all data provided in this study are ecological data. However, these are the most reliable data available to compare the regional difference in NCD burdens at the provincial level. Second, some data, e.g., the prevalence of obesity, hypertension, and diabetes by provinces, were not updated or current due to limited published data. Third, no time trend on NCD burdens for the selected cases was reported due to limited published studies. Fourth, this study used data from different years for investigating multiple conditions. This constraint was due to the unavailability of public comparable data among cases in the same year, particularly for the PG. However, the efforts for such comparison are also important for public health interventions and further research.

### 4.4. Public health implications and recommendations for future work

This study had important public health implications. First, the disparity in data availability revealed in this study suggested the resource disparity in areas with different development levels. These data gaps reciprocally reinforce the current health disparity situation due to limited evidence and public health actions. Second, the narrowing gap in the number of health personnel and health institutional beds between groups suggested significant progress in medical infrastructure in the past decade in China. The remaining overall level of NCD disparities implicates the potential gap in other soft determinants and related actions needed, e.g., high-quality healthcare and improved health literacy level. Third, some traditional dietary features on the Plateau, such as the high consumption of cereals, and low consumption of vegetables and fruits, which may be related to difficulties in accessibility in the past, need to be corrected when the accessibility issue is resolved.

From the research perspective, we have some recommendations. First, more high-quality studies for basic health data collection are required in the less developed areas in China. The severe disparity in data availability suggests that urgent actions are needed. Local research personnel training and research collaboration with developed areas are potential approaches and can be complementary to each other. Meanwhile, health data derived from the national surveillance system should be made available to researchers, thus accumulating intellectual resources to examine and propose solutions to existing health disparity problems. Second, the concepts and methodologies of system science and implementation science are required to generate feasible and effective multidisciplinary approaches (60). Third, culturally adapted intervention studies are needed in ethnic minority populations to promote health equity.

From the intervention perspective, increased activities should be considered. First, promoting regional balanced development and reducing socioeconomic disparities are perhaps the root to cure the regional NCD disparities. Second, more resources should be allocated to NCD management in less developed areas, wherein most cases of communicable diseases were prioritized. Third, increasing the number and quality of healthcare personnel in the PG, particularly the quality of primary healthcare (32), may be one of the solutions. Enhancing the quality of training for healthcare personnel, particularly for primary healthcare personnel, is important. Fourth, the integration of public health and clinical services will help in integrating prevention and treatment. Fifth, the utilization of new technology, such as mHealth, in NCD management, may help improve the accessibility to high-quality healthcare for the residents in remote areas (32).

## 5. Conclusion

China has large disparities in NCD burdens. The NCD burdens were much higher among residents on the Plateau than those in megacities. Both socioeconomic disparity and unbalanced diets were postulated to contribute to NCD and health disparity. Integrated policies and actions are needed to decrease disparities in China.

## Author contributions

WP, YR, and YW: designed the research. WJ, TL, and XT: literature search. WP and WJ: writing—original draft preparation. MM, MH, YW, and YR: writing—reviewing and editing. YR and WP had final primary responsibility for the final contents. All authors have read and agreed to the published version of the manuscript.
